# Supplementary material for: Theranostics of Primary Prostate Cancer: Beyond PSMA and GRP-R
Source: Cancers (Basel). 2023 Apr 18;15(8):2345. doi: 10.3390/cancers15082345 (PMC10137308; doi:10.3390/cancers15082345)
Supplement: Supplementary file 1 [file cancers-15-02345-s001.zip › cancers-2258949-supplementary.pdf]

Supplementary material

Theranostics of primary prostate cancer: beyond PSMA and GRP-R

Romain Schollhammer[1,2], Marie-Laure Quintyn Ranty[3], Henri de Clermont Gallerande[1,2], Florine Cavelier[4], Ibai Valverde[5], Delphine Vimont[2], Elif Hindié[1,2,6], Clément Morgat[1,2].

[1]Nuclear Medicine Department, Bordeaux University Hospital, Bordeaux, France.

[2]INCIA, University of Bordeaux, CNRS, EPHE, UMR 5287, Bordeaux, France.

[3]Department of Pathology, University Hospital of Toulouse, 31000 Toulouse, France

[4]Institut des Biomolécules Max Mousseron IBMM, UMR 5247, CNRS, Université Montpellier, ENSCM, Pôle Chimie Balard, 1919, route de Mende, 34093, Montpellier Cedex 5, France.

[5]Institut de Chimie Moléculaire de l'Université de Bourgogne, UMR 6302, CNRS, Université Bourgogne Franche-Comté, 9 Avenue Alain Savary, 21000 Dijon, France.

[6]Institut Universitaire de France (IUF).

Material and method

Radiosynthesis and quality controls of radioligands

All precursors were radiolabeled with ^{111}In, with the exception of pentixafor which was radiolabeled with ^{67}Ga because of the weak affinity of [^{111}In]In-pentixafor towards CXCR4. The general methodology for radiolabeling with ^{111}In and ^{67}Ga was as follow: 50µg (10µg for PSMA-617) of the respective precursors were added to 1.96mL acetate buffer 0.1M pH 4.6 and heated at 90°C for 30 min with commercial $^{111}InCl_3$ or $^{67}GaCl_3$ (Curium Pharma). The raw solution was then purified using a C_{18} cartridge. The final product was then eluted in ethanol and formulated in PBS. Radiochemical purity was checked using radio-UV HPLC and TLC (mobile phase citrate buffer). [^{177}Lu]Lu-DOTATATE was obtained ready-to-use (Lutathera, Novartis/AAA).
